# Association between corrected QT interval and long-term cardiovascular outcomes in elderly patients who had undergone endovascular therapy for lower extremity arterial disease

**DOI:** 10.3389/fcvm.2023.1103520

**Published:** 2023-05-12

**Authors:** Yao-Ting Chang, I-Shiang Tzeng, Shih-Jung Jang, Kuan-Liang Liu, Chien-An Hsieh, Hsin-Hua Chou, Kuan-Hung Yeh, Hsuan-Li Huang, TRENDPAD Study Group

**Affiliations:** ^1^ Division of Cardiology, Department of Internal Medicine, Taipei Tzu Chi Hospital, Buddhist Tzu Chi Medical Foundation, New Taipei, Taiwan; ^2^ Department of Research, Taipei Tzu Chi Hospital, Buddhist Tzu Chi Medical, Foundation, New Taipei, Taiwan; ^3^ School of Medicine, Tzu Chi University, Hualien, Taiwan; ^4^ School of Post-Baccalaureate Chinese Medicine, Tzu Chi University, Hualien, Taiwan

**Keywords:** corrected QT interval, mortality, old aged, low-extremity artery disease, endovascular therapy, major adverse cardiovascular events

## Abstract

**Background:**

Population-based studies have reported the association between prolonged corrected QT (QTc) intervals and an increased risk of adverse cardiovascular events. Data regarding the association between longer QTc intervals and incident cardiovascular outcomes in patients with lower extremity arterial disease (LEAD) are scarce.

**Objective:**

To examine the impact of QTc interval on long-term cardiovascular outcomes in elderly patients with symptomatic LEAD.

**Methods:**

This cohort study extracted data from the Tzu-chi Registry of ENDovascular Intervention for Peripheral Artery Disease (TRENDPAD) and enrolled 504 patients aged ≥ 70 treated with endovascular therapy for atherosclerotic LEAD from July 1, 2005, to December 31, 2019. The main outcomes of interest were all-cause mortality and major adverse cardiovascular events (MACE). Multivariate analysis was conducted using the Cox proportional hazard model to determine independent variables. We performed interaction analysis between corrected QT and other covariates and Kaplan-Meier analysis to compare the outcome of interest among the groups stratified by the tercile of QTc intervals.

**Results:**

A total of 504 patients [235 men (46.6%); mean age, 79.9 ± 6.2 years; mean QTc interval, 459 ± 33 msec] entered the final data analysis. We categorized the baseline patient characteristics according to terciles of QTc intervals. During the median follow-up time of 3.15 (interquartile ranges, 1.65–5.42) years, we noted 264 deaths and 145 MACEs. The 5-year rates of freedom from all-cause mortality (71% vs. 57% vs. 31%, *P *< 0.001) and MACEs (83% vs. 67% vs. 46%, *P *< 0.001) were significantly different among the tercile groups. Multivariate analysis showed that a 1-SD increase in the QTc interval increased the risk of all-cause mortality [hazard ratio (HR) 1.49, *P *< 0.001] and MACEs (HR 1.59, *P *< 0.001) after adjusting for other covariates. The interaction analysis showed that QTc interval and C-reactive protein levels were most strongly associated with death (HR = 4.88, 95% CI 3.09–7.73, interaction *P* < 0.001) and MACEs (HR = 7.83, 95% CI 4.14–14.79, interaction *P* < 0.001).

**Conclusions:**

In elderly patients with symptomatic atherosclerotic LEAD, a prolonged QTc interval is associated with advanced limb ischemia, multiple medical comorbidities, increased risk of MACEs, and all-cause mortality.

## Introduction

With the aging of the worldwide population and advances in medical care, physicians face an ever-growing number of elderly patients with advanced atherosclerotic lower extremity arterial disease (LEAD) (1, 2). Patients with LEAD have a 3- to 4-fold risk of incident cardiovascular (CV) events, even asymptomatic patients (3). Symptomatic patients have a higher risk of subsequent CV events, ranging from 20% for 5-year non-lethal CV events in claudication to 10% for 1-year deadly events in patients with chronic limb-threatening ischemia (CLTI) (4, 5), especially in elderly patients with multiple comorbidities (6–8).

Corrected QT interval (QTc) varies depending on gender and age, with longer QTc intervals in older people and women (9). Population-based studies have shown the association between CV events and death with longer QTc intervals in healthy populations (10–12) and patients with increased CV risk (13, 14). For atherosclerotic LEAD, limited reports have shown the impact of longer QTc intervals on mortality or non-fatal CV events in patients with diabetic foot ulcers (15, 16). The role of longer QTc intervals in elderly patients with symptomatic LEAD has not yet been elucidated. This study aimed to examine the impact of QTc intervals on long-term CV outcomes in older people with symptomatic atherosclerotic LEAD.

## Materials and methods

### Study design and patients

**Figure 1:**
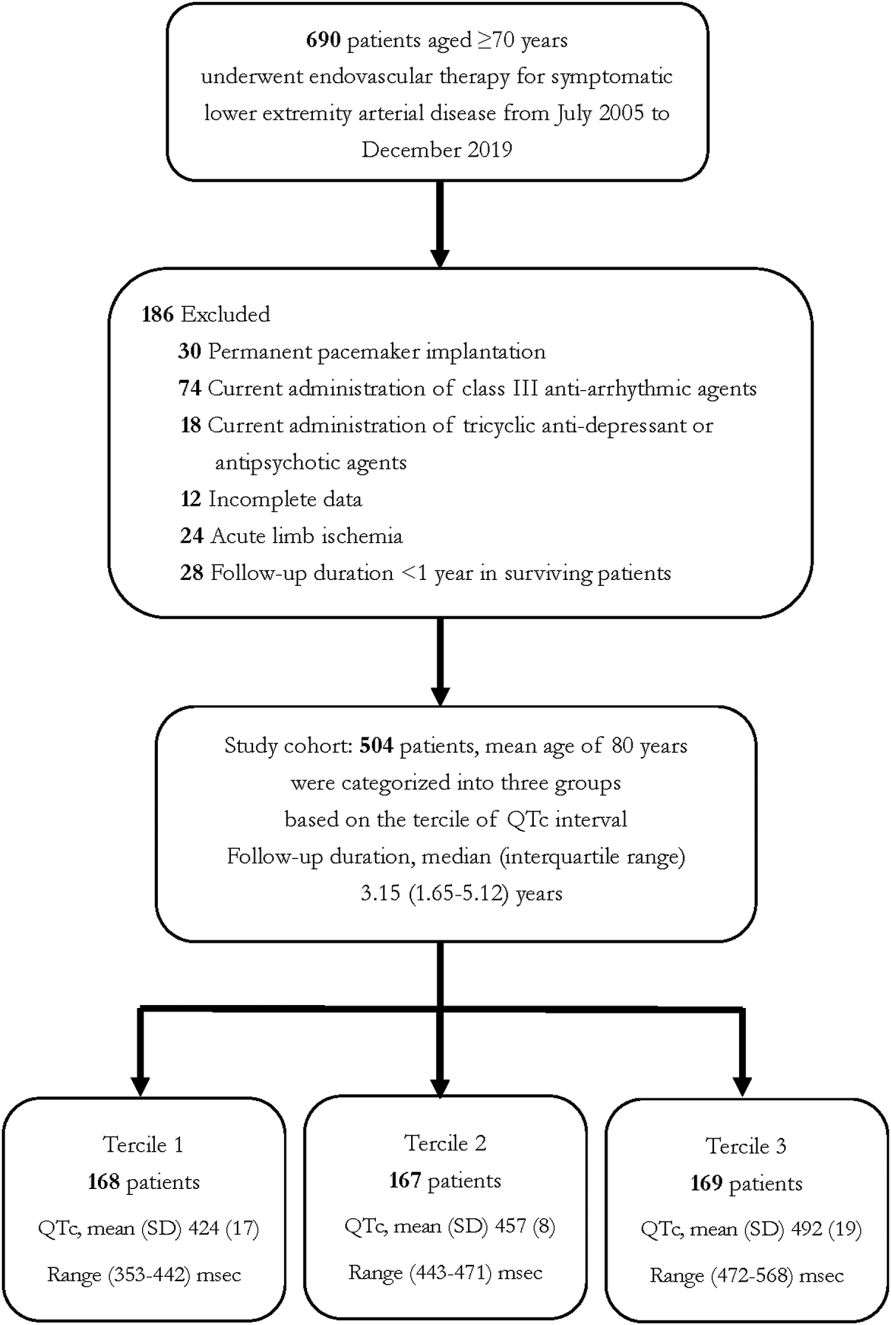
Flow chart of study participants.

We extracted patient-level data from the Tzuchi Registry of ENDovascular Intervention for Peripheral Artery Disease (TRENDPAD), a physician-initiated, prospective single-center observational registry of patients who had undergone endovascular therapy (EVT) for symptomatic LEAD. We enrolled 504 patients aged ≥ 70 who received EVT for atherosclerotic LEAD between July 2005 and December 2019. Patients with acute limb ischemia, non-atherosclerotic LEAD, uncontrollable foot infection, and follow-up duration of less than 1 year were excluded from the study. To avoid factors influencing the measurement of QTc intervals, we excluded patients with permanent pacemaker implantation and those currently receiving class III anti-arrhythmic agents (such as amiodarone, sotalol, or dronedarone), anti-microbial agents (fluoroquinolone, macrolides, and antifungal agents), selected tricyclic antidepressants, or antipsychotics. We categorized study participants into three groups based on the tercile of QTc intervals. Figure 1 depicts the patient enrollment in this study.

We thoroughly reviewed the medical history and records of all patients. Coronary artery disease (CAD) was diagnosed with at least one-vessel stenosis of >50% by previous coronary angiography or a history of myocardial infarction. We defined heart failure with reduced ejection fraction (HFrEF) as a left ventricular ejection fraction <40% based on echocardiography routinely conducted for patients 3 months before the index EVT. A cerebrovascular accident (CVA) was defined as any preceding history of stroke. Patients underwent the scheduled follow-up protocol, including ankle-brachial pressure index, toe pressure index, or duplex ultrasound, 1 week, 1 month, and subsequently every 3 months after the index EVT. This study complied with the Declaration of Helsinki and was approved by the institutional review board of Taipei Tzu Chi Hospital (09-X-067). We obtained written informed consent from all study participants.

### Electrocardiogram (ECG)

All patients received a standard 12-lead ECG at admission using a MAC 5,500 machine (GE Medical System, Milwaukee, WI, USA). The QT interval was measured from the onset of the QRS complex to the end of the T-wave with 3–5 beats in all 12 leads. We averaged the QT interval over 10 s in patients with atrial fibrillation. We adjusted the QT interval for mean heart rate using the Bazett formula (QTc = QT/√RR).

### Clinical outcomes

The main outcome of interest was all-cause deaths and major adverse cardiovascular events (MACEs). MACE comprises cardiovascular death, non-fatal myocardial infarction, and non-fatal stroke. We ascertained death events through a review of hospital records, reports from the next of kin, and retrieval of death certificates. CV death incorporated sudden cardiac arrest or death caused by myocardial infarction, stroke, decompensated heart failure, lethal arrhythmia, valvular heart disease, and aortic or other vascular diseases. For main outcomes, we censored patients at the last contact, date of decease, CV events, or end of the follow-up period. The closing date of the study was December 31, 2021.

## Statistical analyses

We presented continuous data with or without a normal distribution as mean ± standard deviation or median and interquartile range, which were compared using one-way ANOVA or the Kruskal–Wallis test. Categorical data were shown as frequencies and percentages and compared using the chi-squared test. We determined the cutoff value of continuous variables for main outcomes using the receiving-operative characteristic curve. We used the Kaplan–Meier method for time-to-event analyses with censoring. We examined the impact of the QTc interval on the main outcome using a multivariate Cox proportional hazards model. The covariates in the multivariate models contained demographic factors (age, sex, current smoking), traditional CV risk factors [body mass index (BMI), dialysis, diabetes mellitus, and hypertension], previous cardiovascular disease history (CAD, HFrEF, CVA, CLTI, and atrial fibrillation), actual laboratory results [white blood cell count, neutrophil-lymphocyte ratio, C-reactive protein (CRP), and serum albumin], and medication (antiplatelet agents, *β*-blockade, renin-angiotensin-aldosterone system inhibitors, and statins). We included one variable per 10 observed events to prevent model overfitting. We also analyzed the interaction between QTc interval and other significant predictors of all-cause mortality and MACEs. The software used for statistical analyses were Statistical Package for the Social Sciences, version 22.0 (SPSS Inc., Chicago, IL, USA), R software (version 3.4.3; The R Foundation for Statistical Computing, Vienna, Austria), and MedCalc® Statistical Software, version 20.110 (MedCalc Software Ltd, Ostend, Belgium). A *P* < 0.05 was considered as a statistical significance.

## Results

A total of 504 patients were entered into data analysis [235 men (46.6%); mean age, 79.9 ± 6.2 years; mean QTc interval, 459 ± 33 msec]. Patients were categorized according to terciles of QTc intervals (Tercile 1: 353–442 msec; Tercile 2: 443–471 msec; Tercile 3: 472–568 msec). Table 1 summarizes the baseline characteristics of the tercile groups. Compared with the other groups, patients in tercile 3 had a higher proportion of low BMI, advanced limb ischemia, and medical comorbidities, such as CAD, HFrEF, and dialysis dependence. We also found that the tercile 3 group had less use of RAASi medication, a lower serum albumin and potassium level, and a higher level of immune-inflammatory markers (CRP, neutrophil-lymphocyte ratio, and lymphocyte count).

**Table 1:** Baseline characteristics according to tertiles of QTc interval^a^.

Characteristic	QTc interval (msec)			*P*-Value
	Tercile 1	Tercile 2	Tercile 3	
	Patients, No (%)			
	*n* = 168	*n* = 167	*n* = 169	
**Demographic data**				
Age, years	80.0 ± 6.1	79.9 ± 6.4	79.8 ± 6.3	0.896
Male sex	86 (51.2)	76 (45.5)	73 (43.2)	0.318
BMI, kg/m^2^	24.1 ± 3.5	24.1 ± 3.9	23.1 ± 3.4	0.015
Smoking	48 (28.6)	53 (31.7)	39 (23.1)	0.200
ABI	0.54 ± 0.18	0.48 ± 0.19	0.46 ± 0.18	0.001
CLTI	121 (72.0)	132 (79.0)	151 (89.3)	<0.001
QTc interval, msec (Range)	424 ± 17 (353–442)	457 ± 8 (443–471)	492 ± 19 (472–568)	<0.001
**Comorbidity**				
Diabetes mellitus	110 (65.5)	108 (64.7)	121 (71.6)	0.334
Hypertension	148 (88.1)	148 (88.6)	149 (88.2)	0.987
Coronary artery disease	53 (31.5)	57 (34.1)	85 (50.3)	0.001
HFrEF	16 (9.5)	16 (9.6)	38 (22.1)	<0.001
Chronic kidney disease	100 (59.5)	120 (71.9)	134 (79.3)	<0.001
Dialysis dependence	17 (10.1)	48 (28.7)	82 (48.5)	<0.001
Cerebrovascular disease	26 (15.5)	37 (22.2)	36 (21.3)	0.245
Atrial fibrillation	26 (15.5)	21 (12.6)	34 (20.1)	0.164
**Medication, *n* (%)**				
Antiplatelet	159 (94.6)	159 (94.6)	158 (92.9)	0.741
Cilostazol	112 (67.9)	121 (75.2)	106 (66.3)	0.180
RAASi	91 (54.2)	82 (49.1)	63 (37.3)	0.006
CCB	87 (52.7)	73 (45.3)	66 (41.3)	0.109
*β*-Blocker	71 (43.0)	78 (48.4)	63 (39.4)	0.257
Statins	54 (32.1)	41 (24.6)	43 (25.4)	0.234
**Laboratory data**				
Potassium, median (IQR), mmol/L	4.0 (3.6, 4.5)	3.9 (3.5, 4.3)	3.8 (3.4, 4.3)	0.007
Albumin, g/dl	3.23 ± 0.67	3.16 ± 0.60	2.91 ± 0.65	<0.001
Total cholesterol, median (IQR) mg/dl	154 (131, 187)	158 (134, 185)	149 (129, 180)	0.255
LDL, median (IQR) mg/dl	90 (73, 114)	93 (72, 116)	84 (70, 106)	0.188
WBC, median (IQR) 1,000/µl	7.285 (5.915, 8.998)	7.150 (5.160, 8.880)	7.500 (5.720, 10.315)	0.612
Hematocrit, median (IQR) %	34.0 (30.3, 38.2)	33.3 (30.4, 36.7)	32.5 (29.0, 36.7)	0.248
Platelet count, median (IQR) 1,000/µl	222 (172, 272)	223 (172, 273)	212 (164, 269)	0.562
NLR, median (IQR)	3.29 (2.13, 5.89)	3.48 (2.38, 5.07)	4.10 (2.60, 5.94)	0.007
PLR, median (IQR)	158 (108, 231)	150 (117, 217)	163 (115, 230)	0.452
Lymphocyte count, median (IQR) 1,000/µl	1.455 (1.087, 1838)	1.401 (1.109, 1.811)	1.269 (0.984, 1.695)	0.020
HbA1c, (%)	6.8 ± 1.7	6.9 ± 1.8	7.0 ± 1.6	0.801
CRP, median (IQR) mg/dl	0.86 (0.18, 3.81)	1.22 (0.31–3.65)	1.92 (0.45, 6.72)	0.014

CCB, calcium channel blocker; CRP, C-reactive protein; LDL, low-density lipoprotein; RAASi, renin-angiotensin-aldosterone system inhibitor; QTc, corrected QT.

^a^ Tercile 1: 353–442 msec; Tercile 2: 443–471 msec; Tercile 3: 472–568 msec.

**Figure 2:**
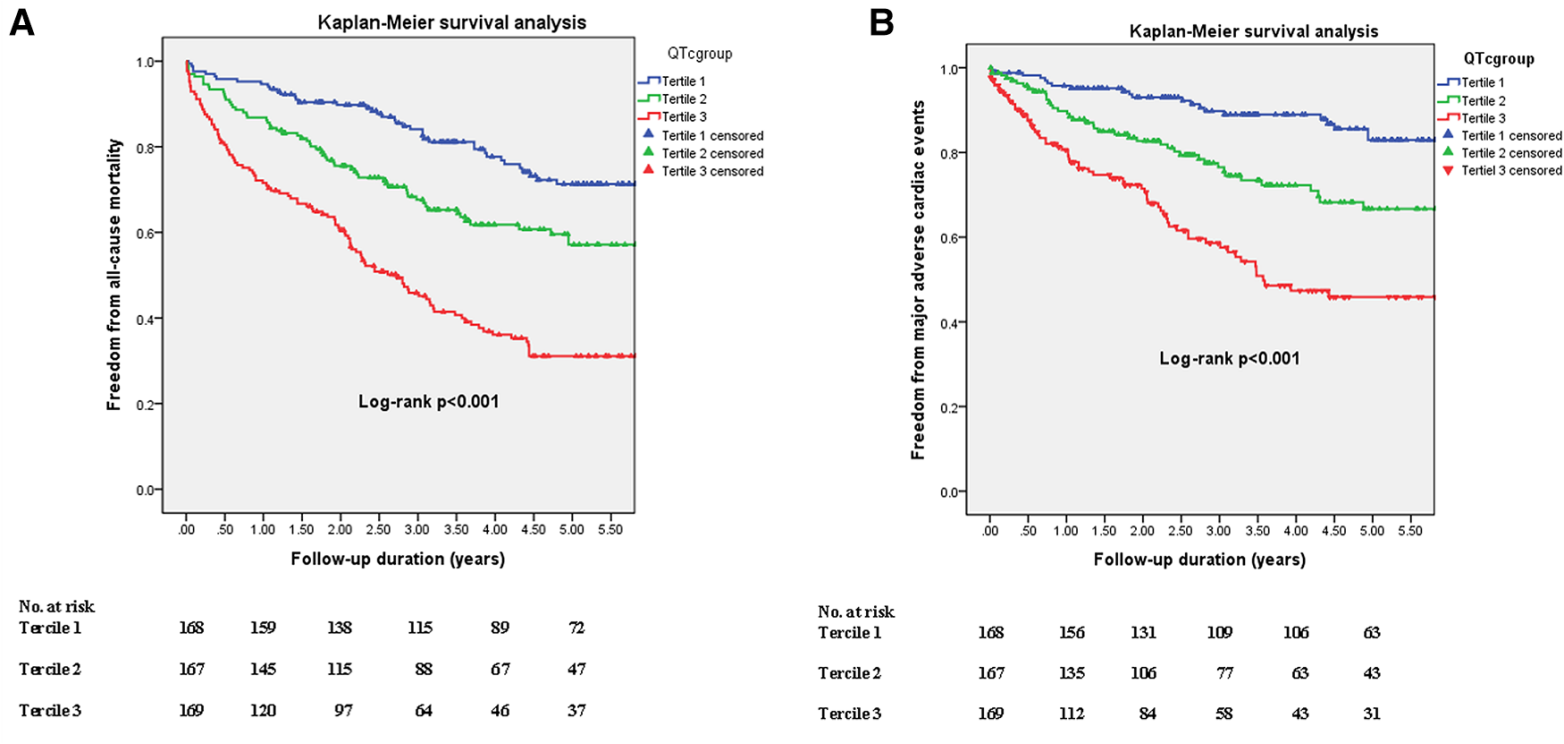
Kaplan-Meier estimates of freedom rates from all-cause mortality (**A**) and major adverse cardiovascular events (**B**) over 5 years in the groups stratified according to corrected QT interval. Blue, green, and red lines denote terciles 1, 2, and 3, respectively.

The median follow-up period was 3.15 (IQR, 1.65–5.42) years, and we noted 264 deaths (183 non-cardiac deaths and 81 cardiac deaths) and 145 MACEs. Most older people die from infectious complications and multi-organ dysfunction, followed by fatal CV events. Eighty-three patients have subsequent coronary interventions during the follow-up period, including 56 acute coronary syndromes and 27 angina pectoris. The 5-year cumulative incidences of following coronary interventions were significantly different among the three tercile groups (12.6%, 17.6%, and 36%, *P *< 0.001) (Supplementary Figure). Kaplan-Meier analysis showed that the 5-year rates of freedom from all-cause mortality (71% vs. 57% vs. 31%, *P *< 0.001) and MACEs (83% vs. 67% vs. 46%, *P *< 0.001) were significantly different among the three tercile groups (Figures 2A,B). Table 2 shows the relationship between QTc interval and clinical outcomes (all-cause mortality and MACE). In univariate analysis, a 1-SD increase in QTc interval increased the unadjusted risk of all-cause mortality (hazard ratio [HR] 1.74, 95% confidence interval [CI] 1.54–1.98, *P *< 0.001) and MACEs (HR 1.20, 95% CI 1.14–1.26, *P *< 0.001) at 5 years. Based on the categorical variables, the risk of all-cause mortality and MACEs increased with a prolonged QTc interval. Compared with tercile 1, the HRs in tercile 3 were 3.81 for death and 4.84 for MACEs (*P *< 0.001). Even after adjusting for clinical and laboratory covariates, the influence of QTc interval on 5-year all-cause mortality and MACEs remained statistically significant.

**Table 2:** Associations between QTc interval and clinical outcomes.

Outcome	Unadjusted		Model 1		Model 2	
	HR (95% CI)	*P* value	HR (95% CI)	*P* value	HR (95% CI)	*P* value
**All-cause mortality at 5 years**						
**QTc, continuous**						
Per SD decrement	1.74 (1.54–1.98)	<.001	1.59 (1.33–1.90)	<.001	1.49 (1.29–1.72)	<.001
**QTc, categorical**						
Tercile 1	Reference		Reference		Reference	
Tercile 2	1.83 (1.23–2.72)	.003	2.08 (1.22–3.55)	.007	1.62 (1.06–2.45)	.025
Tercile 3	3.81 (2.64–5.48)	<.001	3.16 (1.88–5.32)	<.001	2.52 (1.69–3.76)	<.001
**MACEs at five years**						
**QTc, continuous**						
Per SD decrement	1.20 (1.14–1.26)	<.001	1.59 (1.33–1.90)	<.001	1.59 (1.33–1.91)	<.001
**QTc, categorical**						
Tercile 1	Reference		Reference		Reference	
Tercile 2	2.37 (1.40–4.02)	.001	2.09 (1.23–3.56)	.007	1.98 (1.14–3.45)	.015
Tercile 3	4.84 (2.96–7.92)	<.001	3.16 (1.88–5.33)	<.001	3.25 (1.92–5.52)	<.001

Model 1 was adjusted for age, dialysis, coronary artery disease, heart failure with reduced ejection fraction (HFrEF), cerebrovascular accident (CVA), chronic limb-threatening ischemia (CLTI), and atrial fibrillation for all-cause mortality; dialysis, coronary artery disease, HErEF, CVA, CLTI, and atrial fibrillation for major adverse cardiac events (MACE).

Model 2 was adjusted for covariates in Model 1: body mass index, renin-angiotensin-aldosterone system inhibitor, stains, white blood cell counts, neutrophil-lymphocyte ratio (NLR), and C-reactive protein (CRP).

Model 2 for MACE: Model 1, hematocrit, NLR, CRP, albumin, and body mass index.

Tercile 1: 353–442 msec; Tercile 2: 443–471 msec; Tercile 3: 472–568 msec.

**Figure 3:**
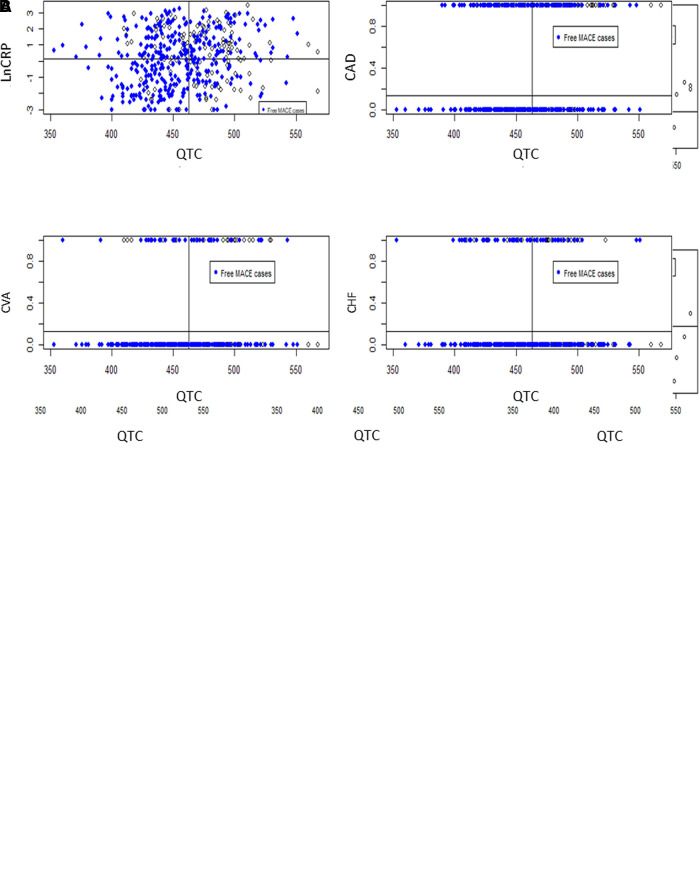
The scatter plot shows the synergistic power in predicting the outcome of interest when combining the QTc intervals with other independent covariates. (**A**) shows the all-cause mortality rate when using the QTc > 465 msec and C-reactive protein (CRP) > 2.1 mg/dl. The other covariates were age > 77.2 years, BMI ≤ 20 kg/m^2^ or serum albumin ≤ 3.0 mg/dl, coronary artery disease (CAD), or cerebrovascular accident (CVA). (**B**) shows the major adverse cardiovascular event rate using the QTc > 463 msec and CRP > 0.88 mg/dl. The other factors were CAD, CVA, or heart failure with reduced ejection fraction.

Table 3 shows the multivariate analysis of the outcome of interest. Age, BMI, CAD, CVA, QTc interval, CRP, and albumin were independent factors for all-cause mortality, and predictors for MACEs were HFrEF, CAD, CVA, QTc interval, and CRP. We performed an interaction analysis between QTc intervals and other significant covariates. The results showed that the QTc interval remained a significant predictor for primary outcomes and that the QTc interval and CRP level were most strongly associated with death (HR = 4.89, 95% CI 3.09–7.73, interaction *P* < 0.001) and MACEs (HR = 7.83, 95% CI 4.14–14.79, interaction *P* < 0.001). The scatter plot (Figures 3A,B) reveals that longer QTc intervals can independently predict the outcome and synergistically affect death and MACEs when combined with other predictors. For example, we found the highest rate of all-cause mortality (77%) in patients with QTc >465 msec combined with CRP level >2.1 mg/dl and MACEs (39%) in patients with QTc >463 msec coupled with CRP >0.88 mg/dl.

**Table 3:** Cox regression analysis for outcome of interest .

Factors	Univariate analysis HR (95% CI)	Unadjusted *P*-value	Multivariate analysis HR (95% CI)	Adjusted *P*-value	Interaction with multivariate analysis HR (95% CI)	Interaction *P*-value
**All-cause mortality**						
Age	1.03 (1.01–1.05)	0.004	1.04 (1.01–1.06)	0.002	4.23 (2.49–7.16)	<0.001
BMI	0.91 (0.88–0.95)	<0.001	0.96 (0.92–1.001)	0.051	4.05 (2.56–6.40)	<0.001
CAD	1.49 (1.16–1.90)	0.001	1.54 (1.12–2.12)	0.008	3.86 (2.61–5.71)	<0.001
CVA	1.69 (1.27–2.25)	<0.001	1.52 (1.07–2.16)	0.019	3.58 (2.15–5.96)	<0.001
QTc interval	1.02 (1.01–1.02)	<0.001	1.01 (1.01–1.02)	<0.001	-	
CRP	1.35 (1.25–1.46)	<0.001	1.17 (1.04–1.32)	0.009	4.89 (3.09–7.73)	<0.001
Albumin	0.43 (0.36–0.52)	<0.001	0.681 (0.50–0.92)	0.014	4.59 (3.07–6.87)	<0.001
**Major adverse cardiovascular events**						
HFrEF	2.62 (1.73–3.95)	<0.001	1.99 (1.16–3.15)	0.003	5.49 (3.28–9.20)	<0.001
CAD	2.03 (1.44–2.87)	<0.001	1.63 (1.08–2.44)	0.019	4.65 (2.85–7.59)	<0.001
CVA	2.38 (1.63–3.47)	<0.001	2.37 (1.55–3.62)	<0.001	6.21 (3.43–11.26)	<0.001
QTc interval	1.020 (1.02–1.03)	<0.001	1.02 (1.01–1.02)	<0.001	-	
CRP	1.30 (1.17–1.46)	<0.001	1.21 (1.04–1.40)	0.012	7.83 (4.14–14.79)	<0.001

BMI, body mass index; CAD, coronary artery disease; CVA, cerebrovascular accident; CI, confidence interval; CRP, C-reactive protein; HR, hazard ratio; HFrEF, heart failure with reduced ejection fraction.

## Discussion

This cohort study is the first to demonstrate the association between longer QTc intervals and a rising risk of incident all-cause deaths and MACEs in older people with symptomatic LEAD. Patients with longer QTc intervals had advanced lower limb ischemia, multiple comorbidities, and impaired nutritional and immune-inflammatory status. After multivariate analysis, we noticed that a longer QTc interval was not only an independent predictor but also had a synergistic predictive power on the primary outcome when combined with other covariates.

The Framingham Heart Study showed a graded correlation between QTc interval and all-cause death, CAD-related death, and sudden cardiac death (17). Previous research has also demonstrated a similar association between QTc interval and stroke or peripheral vascular disease (10, 18). Patients with symptomatic LEAD, from disabling claudication to CLTI, carry a higher risk of death and CV events (4, 5), especially elderly patients due to multiple comorbidities, frailty, and poor candidacy for revascularization. Previous studies have shown that older age, CAD, atrial fibrillation, dialysis, CLTI, stroke, malnutrition, non-ambulance use, and a higher neutrophil-lymphocyte ratio are potential predictors of subsequent death and cardiovascular events in elderly patients after revascularization for LEAD (19–21). Kula et al. also reported a strong association of high-sensitivity troponin T and N-terminal-pro-B-type natriuretic peptide with QTc prolongation in patients with chronic kidney disease (CKD). They proposed the crucial role of myocardial ischemia and volume overload in conduction delay and future MACEs in those patients (22). Compared to the tercile 1 group, our tercile 2 and 3 groups have a higher proportion of CKD, dialysis dependence, HFrEF, and CAD, which implied the association of QTc prolongation with myocardial stretch and ischemia. And thus, the tercile 1 group has better survival and fewer MACEs.

Data regarding the association between QTc interval and symptomatic LEAD are scarce. Fragher et al. reported that QTc interval prolongation (>440 ms) increased mortality in 70 diabetic patients who underwent above-the-ankle amputation. The 3-year all-cause mortality rate was 54% in their study (23). Another study also revealed that a longer QTc interval (>440 msec) combined with a lower HbA1c level (<7.5%) could forecast a higher all-cause mortality in 240 patients with diabetic foot ulcers (15). Wang et al. collected data from 331 patients with foot ulcers with an average 4-year follow-up. They found that QTc prolongation independently predicted cardiac death (HR 5.465, *P *= 0.039) but not ulcer healing or recurrence (16).

However, the role of QTc in elderly patients has remained unclear. In this study, we disclosed that a longer QTc interval was strongly associated with all-cause death and MACEs; either QTc interval was used as a continuous variable or tercile categorization. The underlying mechanism between QTc prolongation and CV events has yet to be elucidated. Cardiac autonomic neuropathy (CAN), a severe complication of diabetes, strongly correlates with a roughly five-fold risk of CV mortality (24). Previous research has reported that CAN could be proposed as an imbalance between sympathetic and parasympathetic activity in the myocardium and lower extremities (10, 25, 26). QTc interval prolongation has been used for CAN assessment and might correlate with autonomic failure in diabetic patients (24, 27). In our study, the tercile 3 group had the most prolonged QTc interval and higher proportions of type 2 DM, CAD, HFrEF, dialysis, advanced limb ischemia, malnutrition, elevated NLR, and CRP. These baseline characteristics are common risk factors for CAN. A longer QT interval might also reflect a reduced repolarization reserve, possibly due to sicker elderly patients with many medical comorbidities. Higher sympathetic tone and profound CAN could accelerate atherosclerosis and further predispose these patients to death and CV events.

Multivariate analysis showed that the QTc interval is independent in predicting death and MACEs. Moreover, interaction analysis and scatter plots revealed a synergistic effect between QTc and other covariates for outcome prediction. QTc interval and CRP level were most strongly associated with death and MACEs. Inflammation is crucial for initiating and developing atherosclerosis and triggers CV events (28). CRP is the widely used inflammatory biomarker linked to underlying atherosclerosis and future CV disease. The association between CRP levels and QTc prolongation has been reported in patients with systemic inflammation or hypertension (29–31). Inflammatory biomarkers, such as interleukin-6, prolong the ventricular myocyte action potential by inhibiting the hERG potassium channel in cardiac myocytes, proposing a direct mechanistic link between QTc prolongation and inflammation (32). Symptomatic LEAD included advanced atherosclerosis and tissue inflammation, which might explain the essential role of combined CRP and prolonged QTc interval in predicting death and MACEs.

### Study limitations

Our study had some limitations. First, it was a retrospective analysis using a prospectively maintained database; thus, we cannot confirm the causality, and remaining confounding may persist despite multivariate adjustment. Second, selection bias was evident because all patients were treated with EVT at a single center, and the EVT strategy depended on the operator's discretion. Third, CRP level and QTc interval are dynamic over time. We only used a single baseline measurement to assess the association between QTc interval, CRP level, and adverse outcomes. However, our study addresses the clinical applicability of QTc measurement in elderly patients with symptomatic LEAD in everyday practice. The QTc interval was calculated using Bazzet's formula, which many researchers have widely used. However, this formula often overcorrects heart rate, which may influence the results. In addition, we did not routinely check serum magnesium, calcium, and arterial blood gas for acidosis or alkalosis in each participant, which might influence the actuarial measurement of QTc. Finally, the QTc interval varied with age, and the appropriate cutoff value of prolonged QTc interval in elderly patients requires further study.

## Conclusion

In elderly patients with symptomatic atherosclerotic LEAD, a prolonged QTc interval is associated with advanced LEAD, multiple medical comorbidities, impaired nutrition, and immune-inflammatory status. QTc interval prolongation increases the risk of future MACEs and all-cause mortality and may be considered in the risk assessment of these patients.

## Data Availability

The raw data supporting the conclusions of this article will be made available by the authors, without undue reservation.
