# Supplementary material for: Association between corrected QT interval and long-term cardiovascular outcomes in elderly patients who had undergone endovascular therapy for lower extremity arterial disease
Source: Front Cardiovasc Med. 2023 May 12;10:1103520. doi: 10.3389/fcvm.2023.1103520 (PMC10213350; doi:10.3389/fcvm.2023.1103520)

**Supplementary Figure Legend**

Kaplan-Meier estimates of cumulative incidences of following coronary intervention at 5 years in the groups stratified according to corrected QT interval. Blue, green, and red lines denote terciles 1, 2, and 3, respectively.


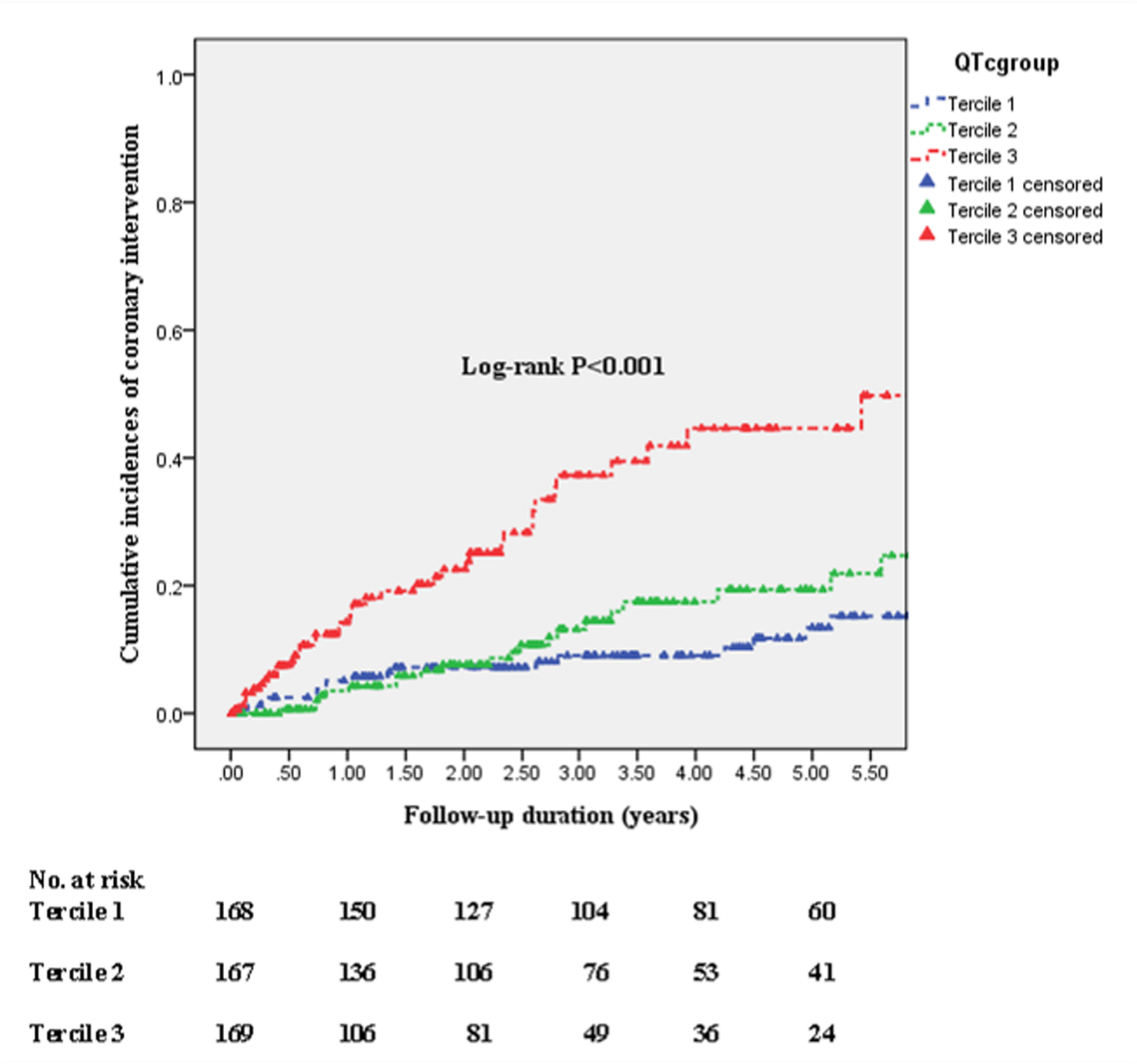

Supplement: Supplementary file 1 [file Datasheet1.docx]
